# Measurement of Inequality and Compression of Mortality in India for 4 Decades: 1970–2015

**DOI:** 10.1093/geroni/igaa057.222

**Published:** 2020-12-16

**Authors:** Mukesh Parmar

**Affiliations:** Jawaharlal Nehru University, New Delhi, India, New Delhi, Delhi, India

## Abstract

The studies relating to measurement of compression of Mortality in India is scarce. Most of the studies relating to mortality in India are focused on either life expectancy, or adult, and child mortality. We have used methods suggested by Kannisto (2000) and Canudos (2008) to measure the compression of mortality phenomenon for India for four decades viz. 1970-2015. Dispersion measures like simple mean, median, modal age at death; and some complicated measures like life disparity, standard deviation above mode, standard deviation in highest quartile, Interquartile range, Gini coefficient, AID and C-family were calculated for India from 1970-2015. We used the age specific death rates from abridged Life tables given by Sample Registration System published by Govt. of India. Our results show that inequality in mortality is decreasing in general but the gap between male and female is increasing. There was an average of three years difference in mean and modal age at death between male females in 2011-15. Overall, mean, median and modal age at death has increased in four decades but other inequality measures like Gini coefficient, AID, Standard deviation (SD) and coefficient of variation has decreased in four decades in India. C50 indicator, which indicates that 50 percent of deaths are happening in that age interval, declined from 26 years to 20 years for males and 27 years to 17 years for females, thus indicating the rate of compression of mortality is higher for females than males in India during 1970-75 till 2011-15.

